# Cranberry juice potentiates sensitivity of uropathogenic *Escherichia coli* (UPEC) strains to fosfomycin and decreases occurrence of spontaneous resistance

**DOI:** 10.1128/aem.02521-25

**Published:** 2026-05-04

**Authors:** Marie-Christine Groleau, Sébastien Houle, Ana C. Quevedo, Geoffrey McKay, Dao Nguyen, Charles M. Dozois, Nathalie Tufenkji, Eric Déziel

**Affiliations:** 1 Centre Armand-Frappier Santé Biotechnologie, Institut National de la Recherche Scientifique (INRS) 14850 , Laval, Québec, Canada; 2 Department of Chemical Engineering, McGill University 5620, Montreal, Québec, Canada; 3 Meakins-Christie Laboratories, Research Institute of the McGill University Health Centre 507266, Montreal, Québec, Canada; 4 Department of Medicine, McGill University 5620, Montreal, Québec, Canada; Michigan State University, East Lansing, Michigan, USA

**Keywords:** fosfomycin, cranberry juice, uropathogenic *Escherichia coli*, antibiotic resistance, sugar transporters, urinary tract infection

## Abstract

Uropathogenic *Escherichia coli* (UPEC) is the leading cause of urinary tract infections (UTIs). The growing prevalence of antimicrobial resistance underscores the need for alternative or complementary strategies to enhance antibiotic activity. Fosfomycin (FOS) remains a recommended first-line treatment for uncomplicated UTIs due to its broad activity and low resistance rates; however, spontaneous resistance frequently arises through mutations in bacterial transport systems. Cranberry juice is known for its anti-adhesive and anti-infective properties; however, its potential to modulate antibiotic activity remains poorly understood. Here, we show that cranberry juice markedly potentiates the antibacterial activity of FOS and limits the emergence of resistance in UPEC clinical isolates. In 72% of the 32 tested isolates, cranberry juice significantly increased FOS inhibition activities and reduced spontaneous FOS-resistant mutant frequencies by up to five orders of magnitude. Whole-genome sequencing revealed distinct mutational patterns: FOS-resistant mutants selected without cranberry juice primarily carried *glpT* mutations, whereas those obtained with juice harbored mutations in *uhpT* or associated regulatory genes. Reporter assays indicated that cranberry juice represses *glpT* expression while maintaining UhpT-mediated FOS uptake, thereby sustaining antibiotic entry and activity. These results demonstrate that cranberry juice alters bacterial carbohydrate transport regulation to potentiate FOS activity and suppress resistance emergence. This study provides novel evidence that a natural product can enhance FOS activity, highlighting its potential as an antibiotic adjuvant for UTI management.

**IMPORTANCE:**

Antimicrobial resistance is a growing threat to public health, and new strategies are needed to preserve the activity of existing antibiotics. This study reveals that cranberry juice, a widely consumed natural product, enhances the antibacterial activity of fosfomycin against uropathogenic *Escherichia coli* by modulating bacterial sugar transport systems. By shifting fosfomycin uptake from GlpT- to UhpT-mediated pathways, cranberry juice both potentiates antibiotic activity and suppresses the emergence of resistant mutants. These findings provide new insight into how dietary components can influence antibiotic response, offering a promising basis for developing natural adjuvants that extend the lifespan of current antimicrobial agents.

## INTRODUCTION

Uropathogenic *Escherichia coli* (UPEC) is the primary etiological agent of community-acquired urinary tract infections (UTIs), contributing to up to 85% of cases (1). In some individuals, recurrent infections following antibiotic treatment are common. Catheter-associated UTIs (CAUTIs) are also a common infection of inpatients in health care facilities, and UPEC represents about 65% of such infections. UTIs can lead to complications, including pyelonephritis and sepsis, renal damage, and chronic health issues due to repetitive use of antibiotics in individuals with recurrent infections. First-line treatment drugs for uncomplicated UTIs in women are frequently trimethoprim-sulfamethoxazole (TMP-SMX), nitrofurantoin, or fosfomycin, with recommended use of fluoroquinolones and beta-lactam antibiotics as secondary agents (2).

Over the past few decades, a growing concern for treatment of UTIs is the increased level of antimicrobial resistance and emergence of multidrug-resistant strains, limiting the options for treatment (3–6). It is therefore of interest to investigate novel therapeutic avenues and means of improving the activity of current antibiotics (7, 8). Alternative approaches to treat UTIs include the development of vaccines and the use of molecules that can potentially block the assembly of *E. coli* fimbrial (pilus) adhesins or molecules that can inhibit adherence of UPEC to host uroepithelial cells. Another approach that has been of keen research interest is the identification of antibiotic adjuvants (9). These products generally exhibit no antimicrobial activity and can either improve the antimicrobial activity of specific antimicrobial agents or suppress the emergence of antimicrobial resistance when given in combination with certain antibiotics.

Fosfomycin (FOS) is an antibacterial phosphoenolpyruvate (PEP) analog used as a single-dose oral treatment for uncomplicated lower UTIs caused by UPEC and *Enterococcus faecalis* (10–12). FOS acts by targeting the first step in bacterial peptidoglycan synthesis mediated by the UDP-GlcNAc enolpyruvyl transferase encoded by *murA*, which is an essential gene (13). FOS has good bactericidal activity against a large range of uropathogenic Gram-positive and Gram-negative bacteria, including extended spectrum beta-lactamase (ESBL)-producing and multidrug-resistant *E. coli* strains. This antibiotic has been used in Japan, South Africa, Brazil, and numerous European countries for over four decades with minimal development of resistance in *E. coli* clinical isolates (14). In North America, treatment of uncomplicated UTIs with FOS began in the late 1990s, after considering its effectiveness as a single-dose treatment due to accumulation in the urinary tract, safety of use in different patient groups, and the low-level of resistance observed in *E. coli* clinical isolates (11).

Despite the low reported levels of FOS resistance in clinical UPEC isolates, the emergence of resistant clones during laboratory culture is very common. Multiple modes of resistance to FOS have been identified, including transmissible plasmid-encoded resistance genes and multiple types of spontaneous or intrinsic mutations that can reduce sensitivity to FOS (10, 11, 15, 16).

Entry of FOS inside the cell occurs through carbohydrate uptake systems. The most common type of resistance observed is due to reduced entry of the antibiotic into the bacterial cell, either by inactivation of the glycerol-3-phosphate transporter (GlpT) or the hexose 6-phosphate uptake transporter (UhpT). The gain in resistance to FOS by decreased uptake can also result in a decrease or loss of growth on specific sugars as a carbon source. Specifically, a marked decrease or lack of growth on glycerol-3-phosphate (G3P) occurs in GlpT-deficient strains, whereas decreased growth with glucose-6-phosphate (G6P), or other hexose phosphates, occurs in UhpT-deficient strains (17–19). Inactivation of the *ackA* and *pta* genes, which are implicated in acetyl-coenzyme A (CoA) degradation, also leads to reduced levels of *glpT* gene expression and a concomitant increase in FOS resistance (20).

Furthermore, decreases in *uhpT* gene expression can also lead to increased FOS resistance. The UhpT transporter is regulated by a phosphorelay system comprised of a DNA-binding protein encoded by *uhpA*, a regulatory kinase encoded by *uhpB,* and a membrane-bound sensor encoded by *uhpC*. Inactivation of any one of these regulatory elements leads to FOS resistance (21, 22). Spontaneous FOS resistance can also occur through reduced expression of the GlpT or UhpT transporters due to mutations affecting the levels of cyclic AMP (cAMP). As such, mutations affecting CRP function (*crp*) or genes related to the control of cAMP levels, such as *ptsI* or *cya* genes, can reduce FOS uptake and sensitivity by decreasing levels of the GlpT and/or UhpT transporters (23–27).

Due to the limited emergence of spontaneous resistance to FOS in UPEC isolates, despite its usage for over 40 years in some countries, it has been suggested that mutations commonly arising *in vitro* may have a high biological cost or *in vivo* disadvantage due to decreased metabolic capacity or fitness (18, 24). However, Bermudez et al. (28) recently determined that some FOS-resistant UPEC isolates with mutations in either *uhp* genes or in pyruvate kinase genes (*pykA* or *pykF*), which play a key role in glycolysis by conversion of PEP to pyruvate, are not attenuated in a murine UTI infection model. Taken together, despite demonstrating good activity and limited emergence of resistance, there is a potential for the generation of FOS resistance in UPEC due to certain types of mutations. This is a cause for concern, since FOS is currently considered a valid choice as an effective treatment for uncomplicated lower UTIs caused by UPEC including multidrug-resistant strains (11, 29–31).

The North American cranberry (*Vaccinium macrocarpon* L.) and its derivatives have long been the focus of investigations on their beneficial effects against UTIs. The primary benefit of cranberries to potentially reduce UTIs was initially linked to the highly acidic nature of cranberries, but later, studies determined that specific components, such as proanthocyanidins and fructose, can inhibit bacterial adherence to uroepithelial cells (32–35). In addition to anti-adherence properties, the activity of cranberry components to potentiate antibiotic activity was reported (36, 37). Considering the high level of spontaneous FOS resistance observed for *E. coli* clinical isolates during laboratory culture, the current use of FOS in several countries and its expected importance as an alternative in patients infected by multidrug-resistant strains in other countries, and the already established benefits of cranberry components against UPEC and UTIs, the goal of this study was to investigate whether exposure of UPEC to pure cranberry juice (CJ) had a potentiation effect on increasing sensitivity of different UPEC clinical isolates to FOS. Not only did we find efficient potentiating activity of CJ, but we also observed an important reduction in the rate of development of resistance to FOS.

## MATERIALS AND METHODS

### Strains and growth conditions

Strains used in this study are listed in Table S1. *E. coli* strains were routinely grown in Lysogeny broth (LB) Lennox (Oxoid) at 37°C. Pure CJ was provided by the Cranberry Institute (USA). Its composition is listed in Table S2.

### Disk diffusion assay

Cation-adjusted Mueller-Hinton II (CAMH-II) plates (150 mm diameter) (Difco), solidified with 1% agar, were prepared with or without a final concentration of CJ of 50% (vol/vol). Briefly, a 2× CAMH-II broth (Difco) containing 2% agar was prepared and mixed at a ratio of 1:1 (vol:vol) with filter-sterilized CJ or water. The pH was adjusted to 6.0 for both conditions (with or without CJ). This pH was chosen to be closer to the pH of urine. Bacterial suspensions were prepared by diluting an overnight culture of bacteria to a 1:100 ratio in CAMH-II broth, pH 6.0, and evenly spread on the surface of the plates. FOS stock solution was prepared at 50 mg/mL in water and filter-sterilized. Different amounts of antibiotics were deposited on blank 7 mm antimicrobial susceptibility disks (Oxoid). Disks were placed on the surface of the freshly inoculated plates, which were then incubated for 16 h at 37°C. Diameters of growth inhibition zones were measured at two different angles, and the surface area was calculated. Pictures of zones were taken using a 6.1× objective with a Leica S9i digital microscope with an integrated 10 MP camera. All plates were prepared in triplicate.

### Frequency of mutant emergence assay

CAMH-II agar plates with or without CJ were prepared as described above, but varying concentrations of FOS were added to the plates. A control plate without antibiotic was prepared to measure the number of bacteria in the initial inoculum. Overnight cultures of *E. coli* were diluted in fresh CAMH-II broth at an OD_600_ of 0.05 and grown until an OD_600_ of 1.0. Serial dilutions were then performed in 0.8% NaCl, and 10 µL of each dilution was spotted on the surface of each plate. After incubation for 24 h at 37°C, resistant colonies were counted (CFU/mL). The frequency of mutant emergence was calculated as the ratio of CFUs with exposure to FOS against the total CFUs determined without any antibiotic. Experiments were performed in triplicate and repeated at least twice.

### Selection of resistant colonies for whole-genome sequencing

Colonies growing inside the inhibition zone around the FOS disks were picked. Colonies at various distances from the disks and obtained after exposure to various concentrations of FOS were selected. Resistant colonies that were able to grow on plates containing FOS were also selected. Each colony was isolated on LB agar, and its resistance profile to FOS was confirmed by a disk diffusion assay on CAMH-II agar using 200 µg of FOS deposited on blank 7 mm disks.

### Genome sequencing and bioinformatic platforms

All whole-genome sequencing (WGS), including library preparation, was carried out by SeqCenter (Pittsburgh, PA). For short-read sequencing, genomic DNA libraries were constructed using the Illumina DNA Prep kit, which employs a tagmentation-based workflow followed by PCR amplification. Libraries were indexed with custom IDT 10-bp unique dual indices and size-selected to achieve an average insert length of approximately 320 bp. Paired-end sequencing (2 × 151 bp) was performed on an Illumina NovaSeq 6000 platform. Raw sequencing data were demultiplexed, quality-filtered, and adapter-trimmed using bcl-convert (v4.1.5).

For long-read sequencing, libraries were prepared without PCR amplification using the Oxford Nanopore Technologies (ONT) Native Barcoding Kit (SQK-NBD114.24) in combination with the NEBNext Companion Module (E7180L), following the manufacturer’s protocols. Sequencing was conducted on ONT MinION Mk1B or GridION instruments equipped with R10.4.1 flow cells, operating in 400-bp per second sequencing mode with a minimum read length threshold of 200 bp. Super accuracy base calling (SUP model), along with demultiplexing and adapter trimming (dna_r10.4.1_e8.2_400bps_modbases_5mc_cg_sup.cfg), was performed using Guppy (v6.4.6).

Bioinformatic analyses were conducted using the Galaxy web platform (https://usegalaxy.org).

For wild-type *E. coli* strains, a hybrid assembly approach was employed combining long-read Nanopore and short-read Illumina data. Nanopore raw reads were processed using Porechop (v0.2.4) with default parameters to remove adapters. Illumina paired-end reads were quality-filtered using Trimmomatic (v0.38.1). Specifically, adapter sequences were removed via ILLUMINACLIP, followed by the removal of leading and trailing bases with a quality score below 20. A 4-base sliding window was utilized to trim reads when the average quality fell below 20, and any remaining reads shorter than 30 bp were discarded.

Hybrid assembly was performed using Unicycler (v0.5.0) under default settings. Assembly quality and continuity were assessed using QUAST (v5.2.0) and visualized via Bandage Image (v2022.09). Resulting contigs were annotated using Prokka (v1.14.6) with default bacterial parameters.

### Variant discovery in fosfomycin-selected mutants

Illumina reads from *E. coli* mutants were processed using the Trimmomatic parameters described above. Processed reads were mapped to their respective wild-type reference genomes using Minimap2 (v2.26). Single-nucleotide polymorphisms (SNPs) and indels were identified using Snippy (v4.6.0), using the FreeBayes (v1.3.2) variant calling engine. To ensure high-confidence variant discovery and the identification of fixed mutations, raw calls were filtered to retain only homozygous alternate genotypes (GT = 1/1) with a minimum Phred-scaled quality score of 100, a read depth of at least 10×, and an alternate allele fraction (AO/DP) of ≥75%. FreeBayes was run with the following minimum thresholds with a base quality of 13, a minimum mapping quality of 30, and a minimum alternate fraction of 0.05.

To ensure the accuracy of the identified variants, all candidate SNPs and indels were manually validated through visual inspection using the Integrative Genomics Viewer (IGV, v2.16.2). For each mutant, the reference genome (FASTA), functional annotations generated by Prokka (GFF3), read alignments (BAM), and variant call files were integrated. This enabled the verification of call quality, assessment of local coverage depth, and confirmation of the genomic context for each mutation relative to the annotated wild-type features.

### Carbohydrate utilization test

Cells from an overnight culture of strains in LB were washed twice with M9 medium (42 mM Na_2_HPO_4_, 22 mM KH_2_PO_4_, 9 mM NaCl, 19 mM NH_4_Cl, 100 mM CaCl_2_, and 1 mM MgSO_4_). Suspensions at an OD_600_ of 0.05 were prepared in M9 that contained either 0.4% (m/vol) G6P, or 0.4% (m/vol) G3P as the sole carbon source. Then, 200 µL of each suspension was distributed into three separate wells of a Honeycomb plate (Growth Curves Ltd.). The plates were incubated inside a Bioscreen C plate reader (Growth Curves Ltd.) at 37°C for 24 h. Measurements with the wideband filter (400–600 nm) were taken every 30 min, following a 30-s agitation.

### Generation of mutants of UPEC strain CFT073

Mutants with deletion of either the *glpT* or *uhpT* genes in UPEC strain CFT073 were generated using procedures developed by Datsenko and Wanner (38) using *glpT-* or *uhpT*-specific primers and plasmids, as described (39).

### Lux reporter assay

To generate promoter fusions as single-copy transcriptional reporters, the promoter regions of the *glpT* or *uhpT* genes were introduced into a luciferase (*lux*) fusion vector and integrated directly into the chromosome at the silent *att*Tn*7* site in the chromosome of strain CFT073, as described previously (40). To measure luminescence of P*glpT*-lux or P*uhpT*-lux, each strain was grown for 17 h on plates of either CAMH-II pH 6.0 or CAMH-II containing 50% CJ at pH 6.0 (CAMH-II-J). After growth at 37°C, three agar plugs were sampled from each plate using the large end of P1000 micropipette tips. Each plug was retrieved aseptically and dropped into microcentrifuge tubes containing 1 mL phosphate-buffered saline. Following a quick vortex step and centrifugation at 200 × *g* for 1 min, the cell suspensions were collected and deposited in 96-well clear microplates. OD_600_ and bioluminescence were measured using a Cytation 3 multimode plate reader (Biotek). Each assay was repeated three times.

### Statistical analyses

Statistical analyses were performed with the R software version 4.0.2 (http://www.R-project.org/) using the base package “Stats” using one-way analysis of variance (ANOVA), *t*-tests, or logistic regressions where specified. Probability values of less than 0.05 were considered significant.

## RESULTS AND DISCUSSION

### Cranberry juice potentiates the activity of fosfomycin and inhibits the emergence of resistance

To verify if CJ could potentiate the antibacterial activity of various antibiotics relevant to the treatment of UTIs (ciprofloxacin, nitrofurantoin, cotrimoxazole, amoxicillin-clavulanic acid, fosfomycin, norfloxacin, cephalexin, and ceftriaxone), a method to compare the sensitivity of a collection of UPEC strains, with and without CJ, was developed. The most consistent and significant potentiating effect was obtained with fosfomycin (FOS), which led to further investigation.

**Fig 1:**
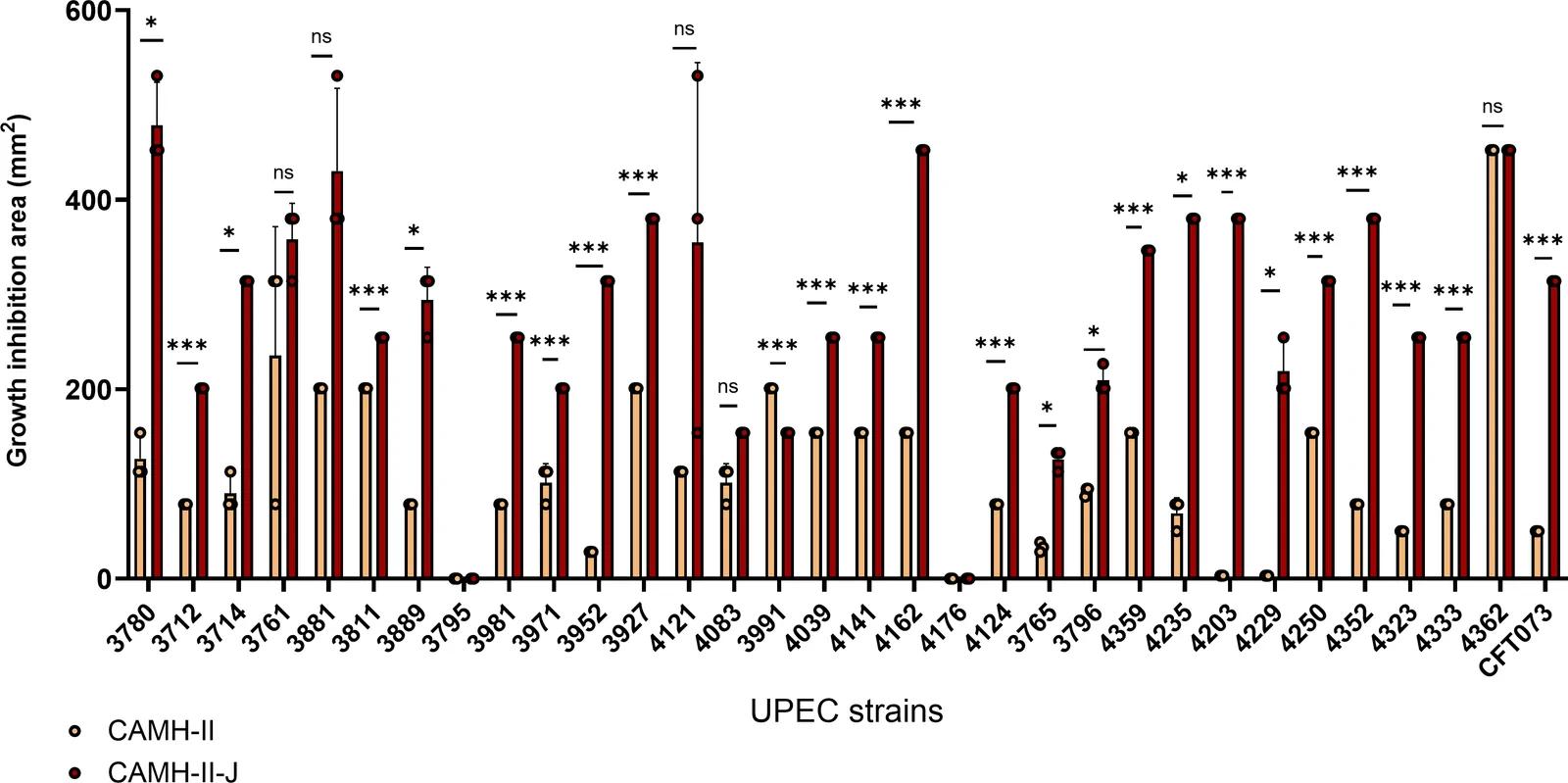
Sensitivity of UPEC strains to FOS with or without cranberry juice. Growth inhibition represented by the area of the inhibition zone (mm^2^) of UPEC strains by FOS with (CAMH-II-J) or without CJ (CAMH-II) was determined using a disk diffusion method. CFT073 is a UPEC type strain. Blank disks were loaded with 16 µg FOS. Standard deviations (error bars) represent variation from three replicates performed on three independent plates. Each dot represents one independent plate. ***, *P* < 0.0005; *, *P* < 0.05; ns, non-significant.

FOS generally shows high activity *in vitro* against UPEC *E. coli*. However, according to the Clinical & Laboratory Standards Institute (CLSI) guidelines, the broth method is unreliable for determining MICs for FOS (14, 41–43), largely due to the spontaneous generation of FOS-resistant cells. We therefore used a disk diffusion assay to determine the potentiating activity of CJ on a panel of 32 *E. coli* isolates from human UTIs, including type strain CFT073. Following the disk diffusion assay to compare inhibition zones on CAMH-II agar with or without 50% juice, we identified a high level of potentiation of FOS by CJ. A significant potentiating activity of CJ on FOS was obtained for 25/32 (78%) of the tested strains (Fig. 1). Among the remaining seven strains, two were completely resistant to FOS (zero inhibition zone at the tested concentrations).

**Fig 2:**
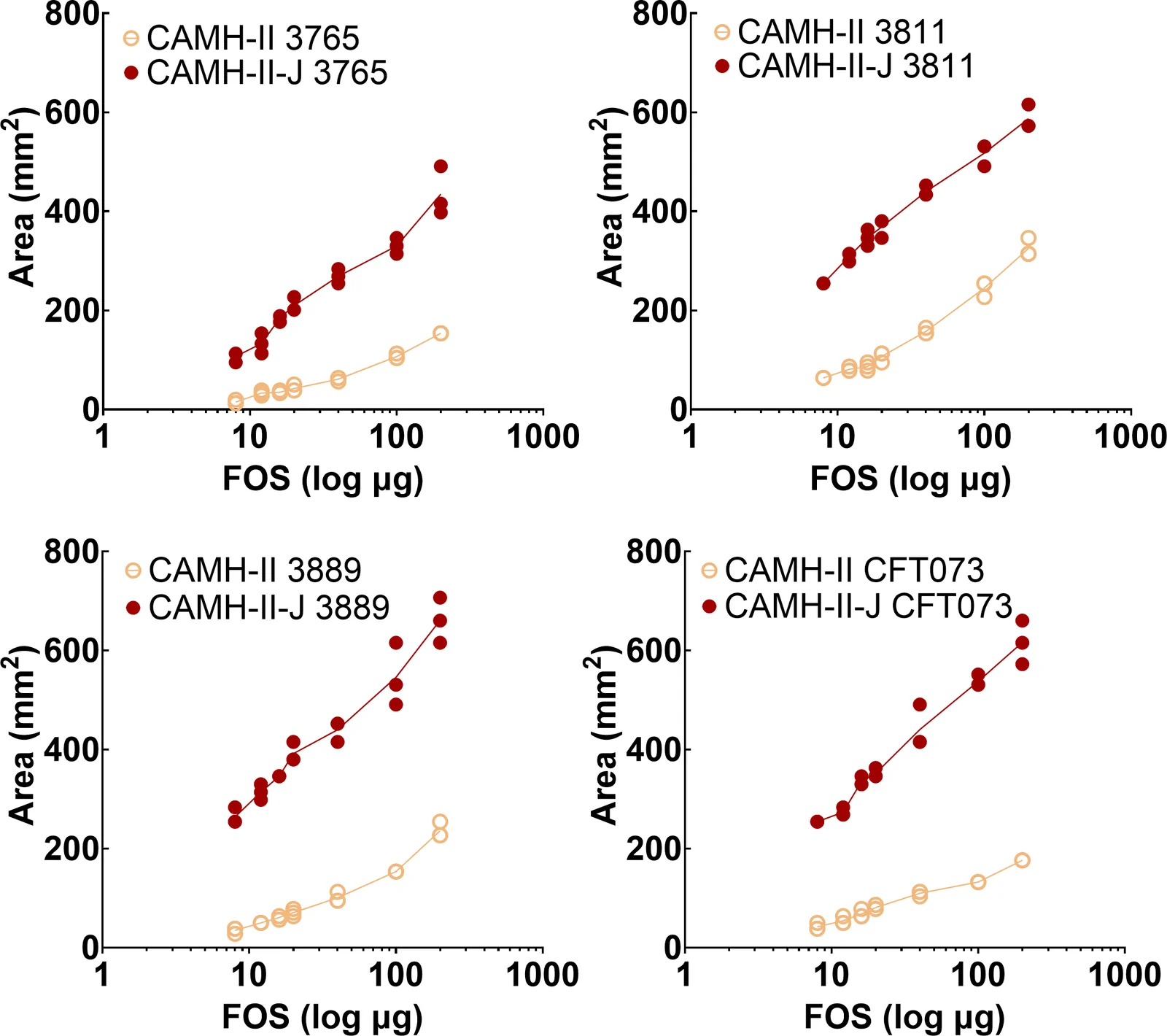
Potentiation of cranberry juice on the growth-inhibiting activity of FOS for *E. coli* strains 3765, 3811, 3889, and CFT073. Graphs present area of the inhibition zones in mm^2^ obtained from disk diffusion assay on cation-adjusted Mueller-Hinton agar with (CAMH-II-J) or without (CAMH-II) 50% CJ. FOS was applied to the disk in amounts ranging from 8 to 200 µg. Each point represents a separate assay.

Next, we tested a larger range of FOS concentrations using the same assay on four selected strains and observed potentiation even at higher concentrations of FOS. Figure 2 presents detailed data for strains 3765, 3811, 3889, and type strain CFT073 exposed to disks with 8–200 µg FOS. For three of the strains, the curves diverge with augmenting FOS concentrations, suggesting increasing potentiation effects.

**Fig 3:**
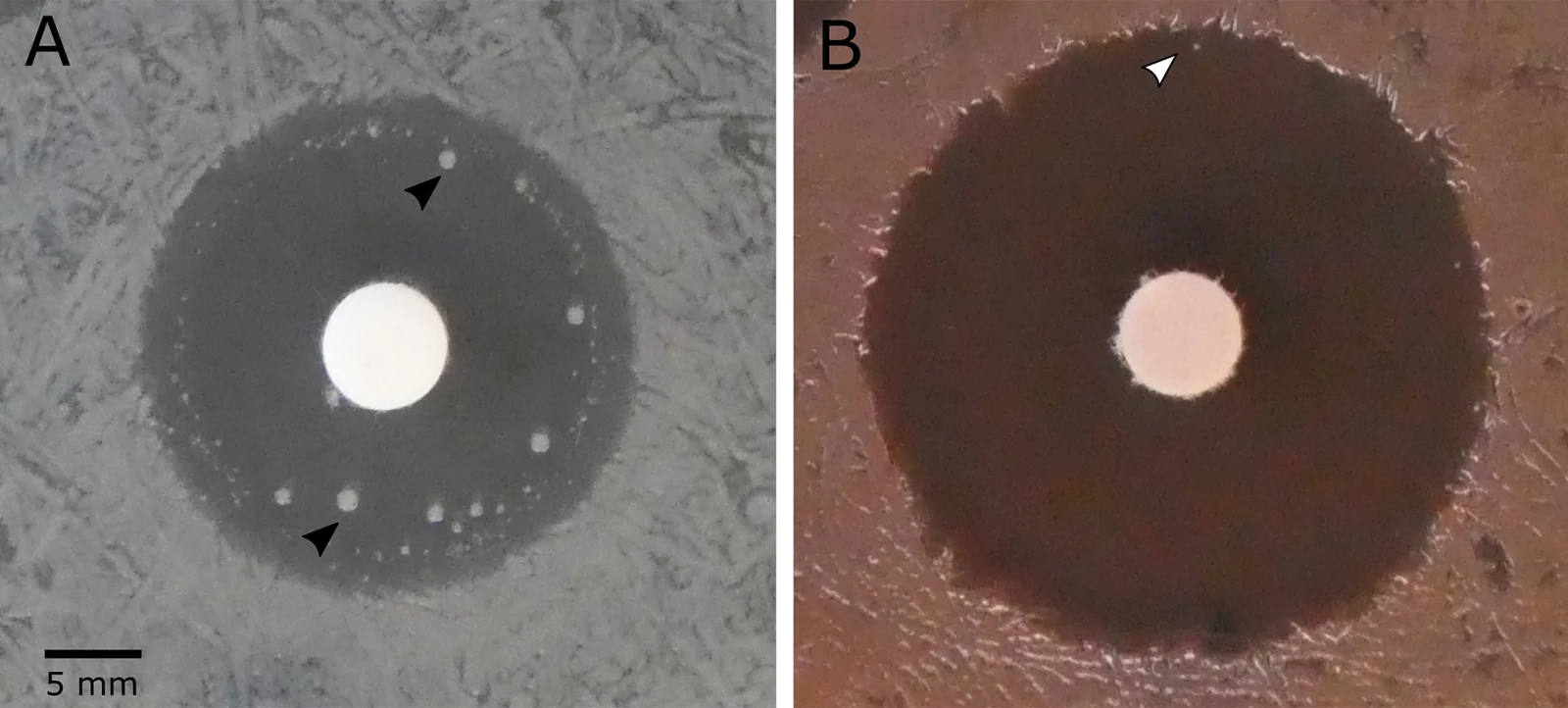
Potentiation of FOS activity by cranberry juice and inhibition of the emergence of colonies inside the inhibition zone**.** A disk containing 200 µg FOS was placed on pH 6.0 CAMH-II (**A**) or CAMH-II-J (**B**) agar plates previously inoculated with UPEC strain 3765. Arrows point to resistant colonies.

Typically, UPEC strains spontaneously develop resistant mutants appearing as small colonies in the growth inhibition zone (42, 44, 45). Resistant colonies inside the inhibition zones were observed for all tested strains except 3795 and 4176, which were completely resistant to FOS, meaning they showed no growth inhibition zones at this concentration. On plates containing CJ, almost no colonies were observed inside the inhibition zones around the antibiotic disks, whereas they were readily visible on plates without juice. An example is presented in Fig. 3 for the UPEC strain 3765.

We also noticed that the few resistant colonies visible within the inhibition zone on plates containing juice were always located farther from the antibiotic disk and appeared smaller. To validate the above observations, we repeated the experiment with another brand of pure organic CJ (Fruits d’Or, Canada) and obtained the same results (data not shown). Importantly, the results were not due to reduced growth on plates containing CJ, as verified by CFU counts from agar plugs recovered from independent plates with and without CJ (Fig. S1). The concentration of bacteria was even slightly higher on plates prepared with CJ, which excludes the possibility that the reduced number of resistant colonies was caused by fewer bacteria on the plate.

**Fig 4:**
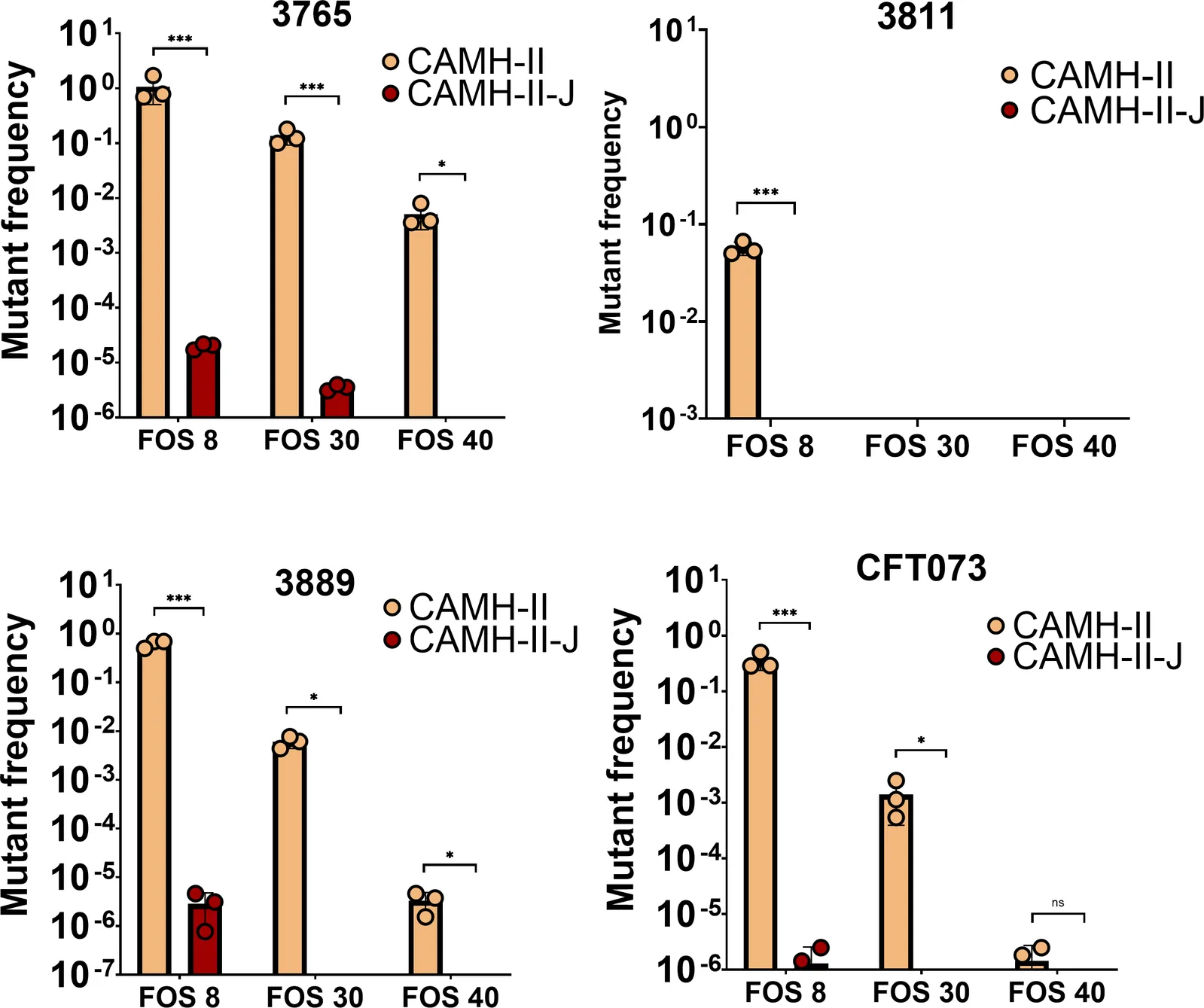
Cranberry juice reduces the frequency of mutant emergence on FOS of four UPEC strains. Serial dilutions of the bacteria were plated on CAMH-II agar containing 50% cranberry juice (CAMH-II-J) compared to CAMH-II agar prepared without juice (CAMH-II). Frequency of mutant emergence represents the ratio between CFUs in the presence of FOS and total CFUs obtained without antibiotics. FOS 8: 8 µg/mL, FOS 30: 30 µg/mL, and FOS 40: 40 µg/mL. A frequency of 10^0^ represents a ratio of 1 or equivalent CFU counts with and without FOS. Each dot represents an independent replicate. All experiments were performed at least twice. Absence of bars indicates absence of growth; this was only observed in the presence of CJ. We tested the impact of CJ on the frequency at which FOS-resistant mutants appeared using logistic regressions. One model was fitted for each concentration of FOS, in which the response variable was the log frequency of mutants’ appearance with and without FOS, and the explanatory variable was the presence or absence of CJ. ***P < 0.001, *P < 0.05, ns: non-significant.

To determine the change in the rate of emergence of resistance to FOS with or without CJ, we performed serial dilutions of cultures of four UPEC strains, inoculated them on plates with inhibitory concentrations of FOS, and compared them with bacterial numbers on plates prepared without the antibiotic. As seen in Fig. 4, there was a marked drop in the rate of spontaneous resistance observed on plates with CJ, whatever the concentrations of FOS tested. Remarkably, the emergence rate dropped by over 5-log (100,000 times) with CJ, and at higher concentrations of FOS (30–40 µg/mL), there was no spontaneous resistance detectable on plates with juice. For instance, when strain 3889 was exposed to 30 µg/mL FOS, the frequency of mutant emergence was less than 10^−7^ (one resistant colony for 10,000,000 total) in the presence of CJ (CAMH-II-J), whereas the frequency of mutant emergence in the absence of juice (CAMH-II) was at least 5 orders of magnitude higher, 10^−2^ (1 resistant colony for 100 total). Furthermore, even with subinhibitory concentrations of FOS, the sensitivity of the strains was enhanced by the presence of CJ (Fig. 4). Strain 3795, which was completely resistant to FOS in our initial assay (Fig. 1), was also tested. It could still grow at concentrations up to 3,000 µg/mL of FOS; remarkably, it became sensitive to FOS in the presence of CJ (Fig. S2).

### Fosfomycin-resistant colonies carry mutations mainly in genes mediating sugar transport

Since a much lower rate of FOS resistance occurred and inhibition zones were also larger in the presence of CJ, we surmised that an increase in the activity of FOS and/or changes in bacterial metabolism in the presence of CJ led to increased sensitivity and decreased emergence of resistant UPEC colonies. To investigate this further, resistant colonies from the four UPEC strains used in Fig. 1 and 2 were re-isolated either from the growth inhibition zones surrounding disks with different concentrations of FOS or from whole plates containing inhibitory levels of FOS. When possible, FOS-resistant colonies that emerged on plates containing CJ (CAMH-II-J) were also selected. Resistance profiles using disks with 200 µg FOS were verified on CAMH-II agar for isolated clones, and FOS-resistant mutants were selected for whole-genome sequencing.

**Fig 5:**
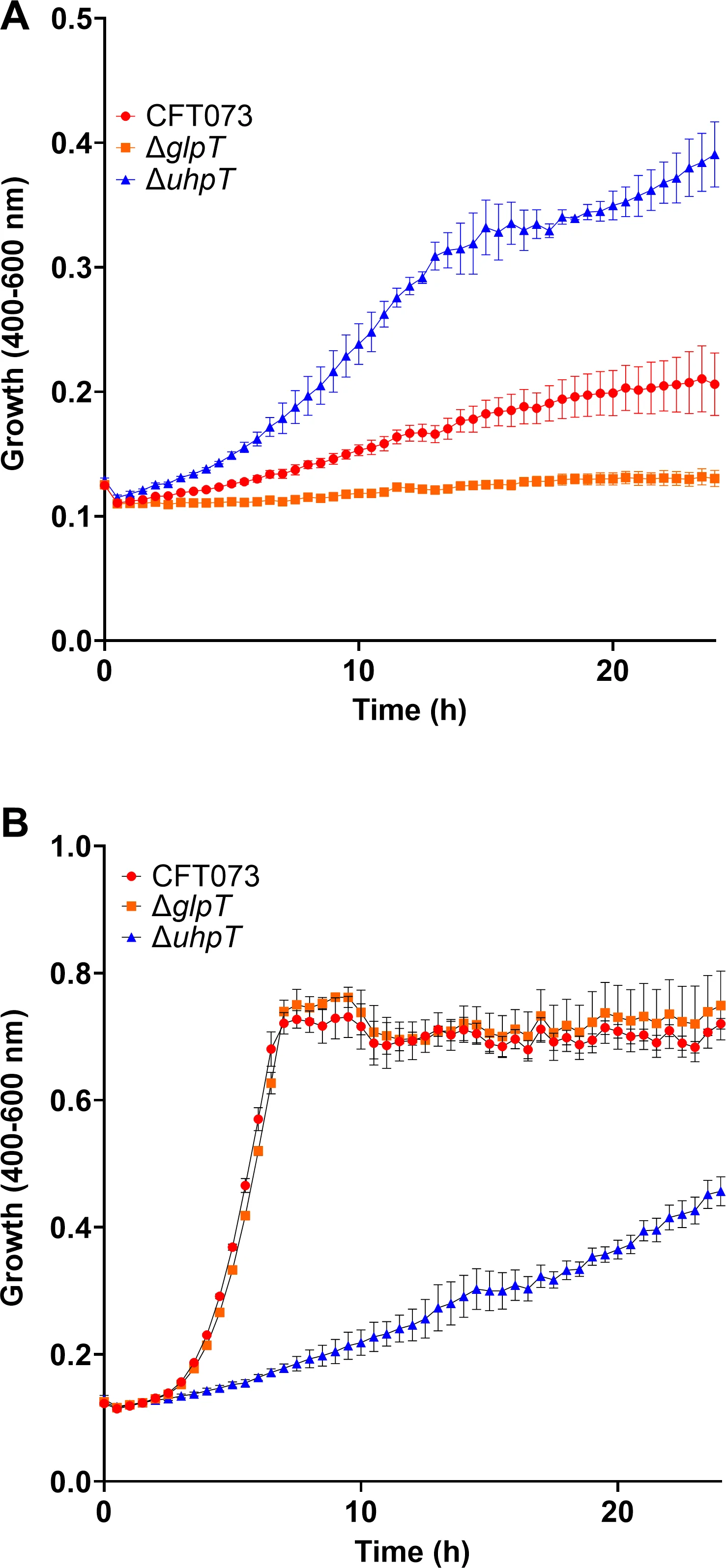
Growth of UPEC strain CFT073 and defined Δ*glpT* and Δ*uhpT* mutants. Bacteria were grown in M9 minimal media with (**A**) 0.4% glycerol-3-phosphate (G3P) or (**B**) 0.4% glucose-6-phosphate (G6P) as the sole carbon source. Growth curves were generated using a Bioscreen C plate incubator and reader with the wideband filter (400–600 nm).

Genome sequencing of these selected resistant clones revealed mutations in the *glpT*, *uhpT*, *uhpC*, *pykF*, or *crp* genes. Interestingly, mutants isolated on plates without juice mostly had mutations in *glpT* encoding the G3P transporter or in the *pykF* gene (Table 1). Resistance profiles of the different clones with mutations specific to *glpT* all had inhibition zones smaller than the parental strain (Table 1; Fig. S3). Mutations in the *pykF* gene were identified in FOS-resistant clones 3765-b, 3811-b, and 3889-c (Table 1). This gene encodes a pyruvate kinase implicated in the conversion of phosphoenolpyruvate to pyruvate during glycolysis (28). In all strain backgrounds, mutations in *glpT* were identified from colonies isolated from CAMH-II plates. On the other hand, among the FOS^R^ colonies isolated from CAMH-II-J plates for sequencing, no *glpT*-specific mutations were identified. These results indicate that mutations in *glpT* are common in UPEC strains exposed to FOS, but rare when exposed to CJ. Instead, out of the six sequenced FOS^R^ clones isolated from plates containing the juice, four had a SNP in genes related to the Uhp transport system. Mutations affecting GlpT can result in an inability of bacteria to grow on G3P. Accordingly, a Δ*glpT* mutant of type strain CFT073 is unable to grow on G3P (Fig. 5). Strains deficient in the Uhp system have a growth defect with G6P as a sole carbon source. The loss of the UhpT system also results in a severe lag in growth on G6P, as shown for Δ*uhpT* in strain CFT073 (Fig. 5). To validate the sequencing results, growth experiments were performed in minimal media with G3P or G6P as a sole carbon source on the sequenced clones that we isolated from strains 3765, 3811, 3889, and type strain CFT073 backgrounds. Conclusions reached based on sequencing and growth experiments for the additional clones are presented in Table 1.

**TABLE 1:** Description and growth profile in G3P or G6P as the sole carbon source of fosfomycin-resistant clones isolated^*a*^

Strain	Zone area (mm^2^)^*b*^	Resistance mutation (from WGS)	Growth profile on:		Predicted affected transport system
			G3P	G6P	
**CFT073**	284.1 ± 29.8		**+**	**+**	
Δ*glpT*	76.2 ± 11.7	*glpT*	−	+	
Δ*uhpT*	218.3 ± 15.0	*uhpT*	+	−	
CFT073-a	56.9 ± 6.7	*glpT*	−	+	GlpT
CFT073-b (J)	259.2 ± 8.3	*uhpT*	+	−	UhpT
CFT073-c	240.7 ± 13.8	*uhpB*	+	+/−	UhpT
CFT073-d	ND	CRP	−	+/−	GlpT
CFT073-e (J)	ND	ND	+	−	UhpT
CFT073-f (J)	ND	ND	+	−	UhpT
CFT073-g	ND	ND	−	−	GlpT/UhpT
**3765**	264.2 ± 16.8		**+**	**+**	
3765-a	22.4 ± 2.4	*glpT*	−	+	GlpT
3765-b	61.6 ± 10.5	*pykF*	+/−	+	
3765-c (J)	ND	ND	+/−	+/−	
3765-d (J)	ND	ND	−	+	GlpT
3765-e (J)	ND	ND	+	+	
3765-f (J)	ND	ND	+	−	UhpT
3765-g (J)	139.67 ± 6.0	*uhpC*	+/−	+	UhpT
3765-h (J)	ND	*topA*_1, *yciK*	+	+	
3765-I (J)	ND	ND	+	+	
3765-j (J)	ND	ND	+/−	+	
**3811**	309.0 ± 9.0		**+**	**+**	
3811-a	101.1 ± 10.4	*glpT/glpA*	−	+	GlpT
3811-b	71.3 ± 13.3	*pykF*	+	+	
3811-c (J)	ND	*trpA* (syn)	+	−	UhpT
					
**3889**	255.4 ± 1.6		**+**	**+**	
3889-a	66.0 ± 4.2	*glpT*	−	+	GlpT
3889-b	ND	ND	+	+	
3889-c	73.4 ± 4.4	*pykF*	+	+	
3889-d (J)	236.1 ± 15.9	*uhpC*	+	+/−	UhpT
3889-e (J)	ND	ND	+	+/−	UhpT
3889-f (J)	192.9 ± 14.1	*uhpC*	+	+/−	UhpT
3889-g	61.6 ± 10.5	*glpT/glpA*, P*uhpA*	−	+/−	GlpT/UhpT

^*a*^ In bold: parental strain. (J): mutant isolated on CAMH-II-J, +: growth, −: no growth, +/−: partial growth, ND: not determined, not sequenced. G3P: glycerol-3-phosphate, and G6P: glucose-6-phosphate.

^*b*^ Area of zones obtained on CAMH-II with disks containing 200 µg of FOS.

To confirm if the presence of CJ specifically selected for resistant mutants in the UhpT system, nine additional resistant colonies picked from inhibitory zones surrounding FOS disks on plates containing CJ were assessed under the same conditions. Indeed, five of them had a growth deficiency on G6P, which points to mutations affecting the function or expression of the UhpT transporter (Table 1). Overall, 9 out of 15 of the very few FOS^R^ clones that could be isolated on CJ during our disk diffusion assays likely have a defect affecting the UhpT system. Loss-of-function mutations in genes related to the UhpT transporter have been detected in colonies appearing in the inhibition zone during antimicrobial susceptibility testing of *E. coli* clinical isolates. Mutations in Uhp components arise in assays performed in conditions where G6P is added together with FOS to induce the Uhp transporter (28, 42, 44, 45). G6P is typically added in standardized antimicrobial testing assays with fosfomycin to increase reproducibility (42, 46). Since we did not add G6P in our assays, we hypothesize that a component in the CJ triggers a similar response.

In the case of *pykF* mutants or some FOS^R^ clones for which no clearly identified base changes linked to FOS resistance were identified and that were not affected in growth with either G3P or G6P, still undefined mechanisms of resistance explain their presence.

### The UhpT transporter is the main route of entry for fosfomycin in the presence of cranberry juice

**Fig 6:**
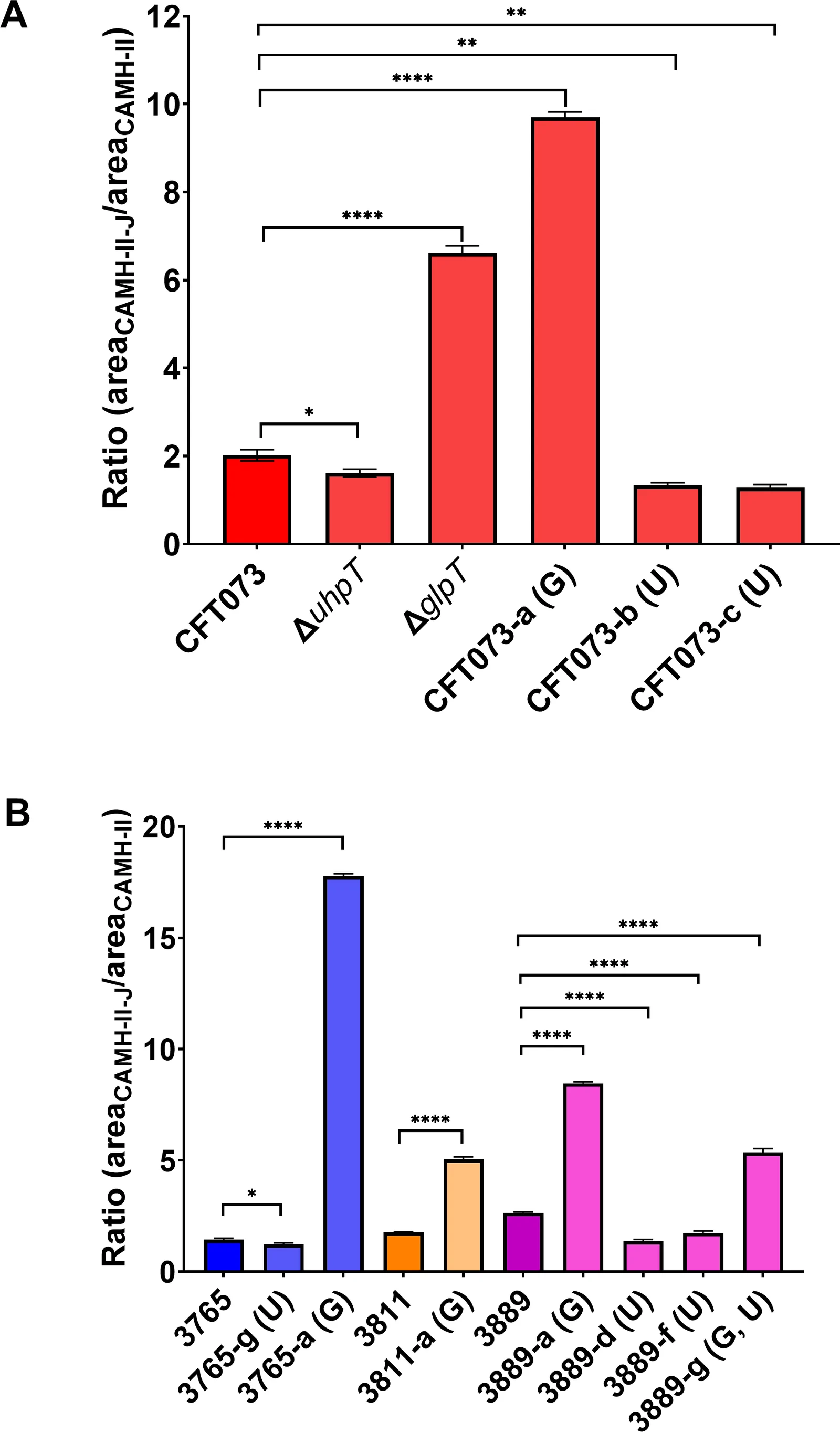
Ratios (fold differences) of the inhibition zone areas obtained on CAMH-II vs. CAMH-II-J agar in disk diffusion assays with 200 µg FOS, after 24 h incubation at 37°C. (**A**) Data obtained for type strain CFT073. (**B**) Data obtained for isolates of strains 3765, 3811, and 3889. Isolates with impaired GlpT system are labeled (G), and isolates with impaired UhpT system are labeled (U). This experiment was performed at least twice with similar results. Error bars were obtained from values of three replicates. ****P < 0.00005, ** *P* < 0.005, *P < 0.05.

Since mutants with a defect affecting the UhpT transport system were mainly obtained on CJ, we postulated that juice promotes switching to entry of FOS primarily using the Uhp transporter, and this might lead to pressure to specifically inactivate or reduce its expression to increase resistance to FOS. To verify this hypothesis, we performed a disk diffusion assay with FOS on *glpT* and *uhpT* deletion mutants of CFT073, in the presence or absence of CJ, to see the impact on FOS activity in these two backgrounds. For the Δ*glpT* mutant, potentiation was even stronger with zones more than 6× larger in the presence of juice (Fig. 6A). Clone CFT073-a, which has a frameshift mutation in the *glpT* gene, showed a similar profile. Since uptake by GlpT is not possible in this background, the uptake of FOS can only be mediated by the Uhp system. This likely indicates that CJ favors the uptake of FOS through the Uhp system, rendering the strain even more sensitive. Accordingly, clones CFT073-b (frameshift mutation in *uhpT*) and CFT073-c (SNP in *uhpB* gene) both had similar profiles as the Δ*uhpT* control strain, with inhibition zones on CJ being smaller than their parental strain, likely due to a lowered uptake of FOS, making these strains more resistant (Fig. 6B; Fig. S3).

When the same experiment was performed using clones from the other tested parental strains (3765, 3889, and 3811), clones with a mutation in genes related to the GlpT system such as 3765-a, 3811-a, and 3889-g were more than 5× more sensitive on CJ, whereas clones with mutations affecting the UhpT system (3765-g, 3889-d, and 3889-f) were more resistant when compared to their parental strain (Fig. 6B; Fig. S3). Taken together, these results validate a model where, in the presence of CJ, the Uhp system is the main transporter responsible for FOS uptake.

### Expression of *glpT* is downregulated in the presence of cranberry juice

**Fig 7:**
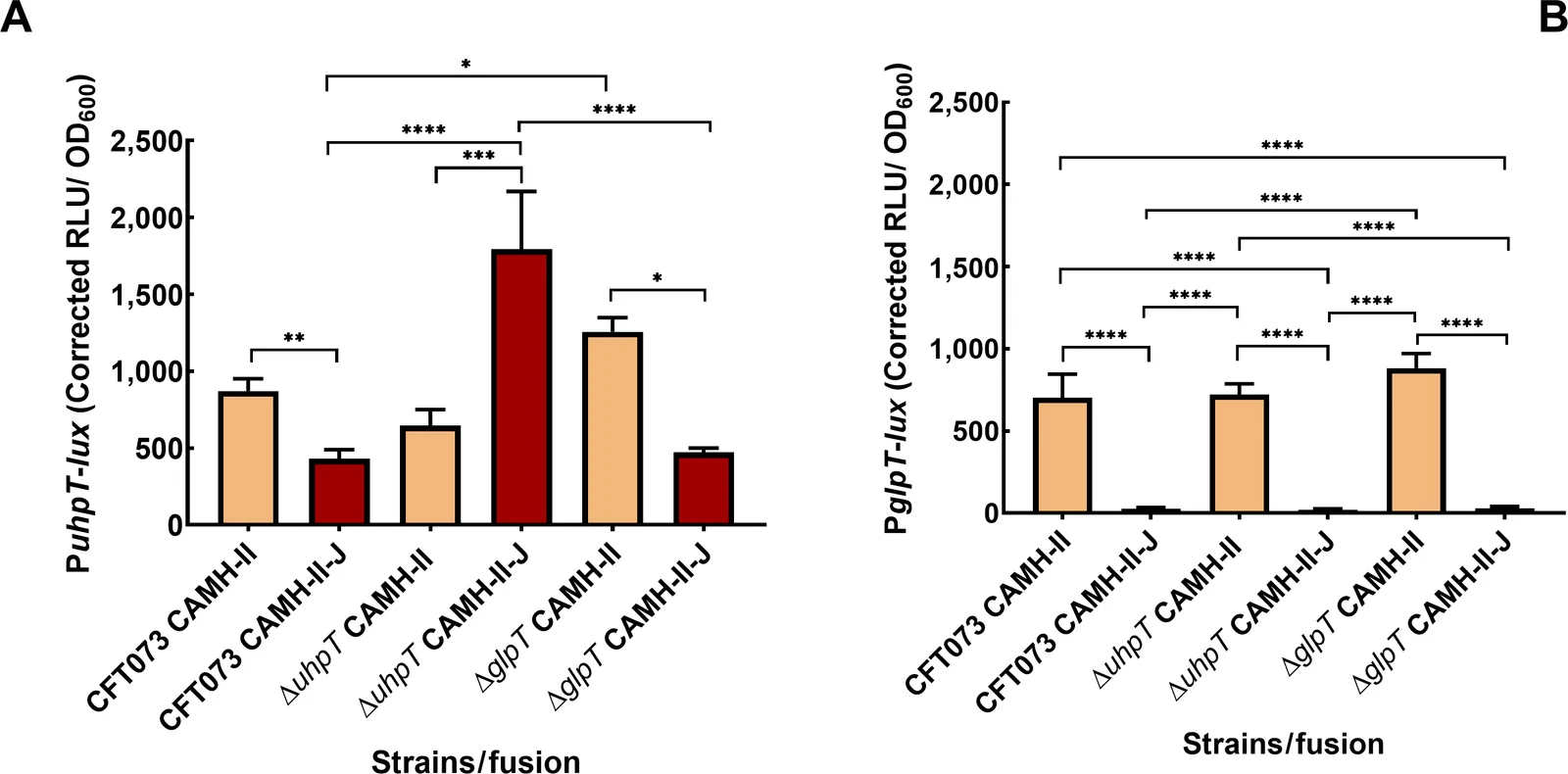
Expression of P*glpT* and P*uhpT* luciferase fusions when grown either in the absence or presence of cranberry juice. (**A**) P*uhpT::lux* expression. (**B**) P*glpT::lux* gene expression. ****P < 0.0001, ***P < 0.0005 **P < 0.005, *P < 0.05.

Since mutants in the *uhp* system are more resistant than their parental strain, this would suggest that the entry of FOS is diminished and thus suggests lower expression of the GlpT system, in this background, in the presence of juice. Differential expression of the GlpT and UhpT systems could explain both the CJ-induced potentiation and different rates of emergence of FOS^R^ colonies. To investigate this, we generated promoter fusions expressing luciferase, which emits light as a quantifiable signal of gene expression from either the *glpT* or *uhpT* promoters, and inserted them in single copy into the genomes of CFT073 and derivatives. These reporter strains were tested on CAMH-II agar with or without CJ, and expression from both promoters was measured in various mutant backgrounds (Fig. 7). The activity of the *uhpT* reporter demonstrated that *uhpT* expression was reduced by CJ in both wild-type CFT073 and its isogenic Δ*glpT* mutant; in the Δ*uhpT* background, activity of the reporter was increased by CJ. Nevertheless, expression from the *uhpT* promoter fusion remained at consistent levels whether in the presence or absence of juice or whether there was a loss of *glpT* or *uhpT* genes (Fig. 7A). By striking contrast, expression of *glpT* is completely abolished in cells exposed to CJ, with expression levels remaining constant and uniform in the absence of juice and not affected by loss of either the UhpT or GlpT systems (Fig. 7B).

We conclude that growth in the presence of CJ essentially shuts down the expression of the GlpT system, normally the main active cell entry mechanism for FOS. In the presence of CJ, only the UhpT system can act as a port of entry for FOS. This correlates well with the lack of a change in the FOS inhibition zones of a WT strain compared to its *glpT* mutant when grown in the presence of juice, as shown in Fig. S3.

The GlpT transporter is repressed by component(s) present in CJ, and the UhpT transporter remains active in the presence of juice, which allows for FOS uptake, leading to higher sensitivity. Mutants unable to activate UhpT-mediated transport in the presence of CJ would uptake less FOS, whereas the sensitivity of resistant mutants in other systems would still be augmented by the presence of the juice. This confirms that the Uhp system is the main entry mechanism for FOS in the presence of CJ.

A number of non-antimicrobial products have been evaluated as potential alternatives to FOS for the treatment of UTIs. However, these agents have not been tested in combination with FOS. Synergistic activity between FOS and various antibiotics has been previously reported (29, 47). To our knowledge, this is the first study to describe a potentiation effect of a natural product—specifically, CJ—on FOS activity for *E. coli*. Previous investigations have examined the influence of cranberry extracts on antibiotic activity and UTI management (48). Potentiation activity of CJ on vancomycin was observed for *Enterobacter cloacae* (49). Although concentrated cranberry extracts rich in proanthocyanidins (PACs) exhibit antimicrobial activity against *E. coli in vitro*, CJ alone does not (50). Moreover, PACs showed no potentiation of FOS for *E. coli* CFT073 (37).

UTIs remain a significant public health concern, particularly considering increasing antimicrobial resistance, which highlights the urgent need for novel therapeutic approaches (50). Recently, the U.S. Food and Drug Administration approved fosfomycin by injection in adult patients with complicated UTIs, including acute pyelonephritis. As the clinical use of this antibiotic expands, it becomes increasingly important to monitor and understand the mechanisms underlying resistance development and its potential mitigation. The findings presented here show that under the *in vitro* conditions tested, a natural dietary product may influence antibiotic activity and bacterial susceptibility. However, these results cannot be directly extrapolated to *in vivo* or clinical contexts. Additional studies are required to determine whether these effects are replicated in physiologically relevant models, to identify the specific component(s) of cranberry juice that are responsible, and to assess whether similar interactions may occur with other antibiotics.

## Data Availability

Sequencing reads were deposited in NCBI under BioProject ID PRJNA1426131.

## SUPPLEMENTAL MATERIAL

Supplemental material aem.02521-25-s0001.pdf Tables S1 and S2; Fig. S1 to S3.

ASM does not own the copyrights to Supplemental Material that may be linked to, or accessed through, an article. The authors have granted ASM a non-exclusive, world-wide license to publish the Supplemental Material files. Please contact the corresponding author directly for reuse.
