# Supplementary material for: Cranberry juice potentiates sensitivity of uropathogenic Escherichia coli (UPEC) strains to fosfomycin and decreases occurrence of spontaneous resistance
Source: Appl Environ Microbiol. 2026 May 4;92(5):e02521-25. doi: 10.1128/aem.02521-25 (PMC13188857; doi:10.1128/aem.02521-25)
Supplement: Supplemental material — Tables S1 and S2; Fig. S1 to S3. [file aem.02521-25-s0001.pdf]

Supplementary material

Table S1: Bacterial strains used in this study

Strains	Description	Source
QT3712	*E. coli* UTI from Guadeloupe, French Antilles	(1)
QT3714	*E. coli* UTI from Guadeloupe, French Antilles	(1)
QT3761	*E. coli* UTI from Guadeloupe, French Antilles	(1)
QT3765	*E. coli* UTI from Guadeloupe, French Antilles	(1)
QT3780	*E. coli* UTI from Guadeloupe, French Antilles	(1)
QT3795	*E. coli* UTI from Guadeloupe, French Antilles	(1)
QT3796	*E. coli* UTI from Guadeloupe, French Antilles	(1)
QT3811	*E. coli* UTI from Guadeloupe, French Antilles	(1)
QT3881	*E. coli* UTI from Guadeloupe, French Antilles	(1)
QT3889	*E. coli* UTI from Guadeloupe, French Antilles	(1)
QT3927	*E. coli* UTI from Guadeloupe, French Antilles	(1)
QT3952	*E. coli* UTI from Guadeloupe, French Antilles	(1)
QT3971	*E. coli* UTI from Guadeloupe, French Antilles	(1)
QT3981	*E. coli* UTI from Guadeloupe, French Antilles	(1)
QT3991	*E. coli* UTI from Guadeloupe, French Antilles	(1)
QT4034	*E. coli* UTI from Guadeloupe, French Antilles	(1)
QT4083	*E. coli* UTI from Guadeloupe, French Antilles	(1)
QT4121	*E. coli* UTI from Guadeloupe, French Antilles	(1)
QT4126	*E. coli* UTI from Guadeloupe, French Antilles	(1)
QT4141	*E. coli* UTI from Guadeloupe, French Antilles	(1)
QT4162	*E. coli* UTI from Guadeloupe, French Antilles	(1)
QT4176	*E. coli* UTI from Guadeloupe, French Antilles	(1)
QT4203	*E. coli* UTI from Guadeloupe, French Antilles	(1)
QT4229	*E. coli* UTI from Guadeloupe, French Antilles	(1)
QT4235	*E. coli* UTI from Guadeloupe, French Antilles	(1)
QT4250	*E. coli* UTI from Guadeloupe, French Antilles	(1)
QT4323	*E. coli* UTI from Guadeloupe, French Antilles	(1)
QT4333	*E. coli* UTI from Guadeloupe, French Antilles	(1)
QT4352	*E. coli* UTI from Guadeloupe, French Antilles	(1)
QT4359	*E. coli* UTI from Guadeloupe, French Antilles	(1)
QT4362	*E. coli* UTI from Guadeloupe, French Antilles	(1)
CFT073	UPEC wild-type pyelonephritis strain (O6:K2:H1)	(2, 3)
CFT073Δ*glpT*	UPEC CFT073 null mutant lacking *glpT* gene	This study
CFT073Δ*uhpT*	UPEC CFT073 null mutant lacking *uhpT* gene	This study
CFT073::P*glpT*	CFT073 with *lux* fusion from P*glpT* promoter	This study
CFT073::P*uhpT*	CFT073 with *lux* fusion from P*uhpT* promoter	This study

Table S2: Composition of the cranberry juice used in this study

Nutrient	Amount (per 100 g)	Unit
Calories	24.59	kcal
Fat	0	g
Saturated fat	0	g
Carbohydrates	7.48	g
Dietary fiber	0.07	g
Soluble fiber	0.07	g
Insoluble fiber	0	g
Total sugars	3.75	g
Dextrose	3.08	g
Fructose	0.65	g
Sucrose	0.02	g
Added sugars	0	g
Protein	0.04	g
Organic acids	7.28	g
pH	2.4	

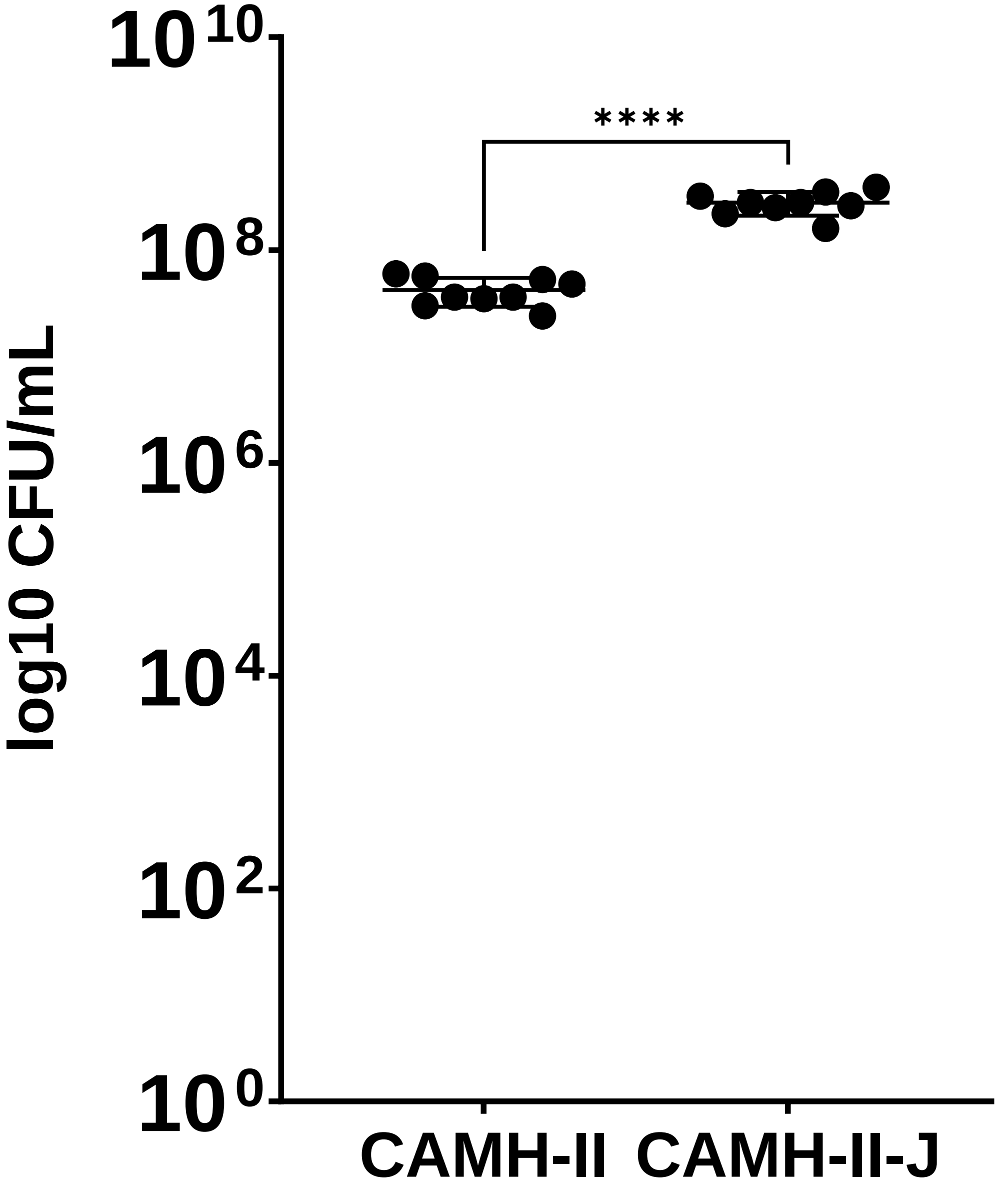

Figure S1: Growth of CFT073 in presence of CAMH-II and CAMH-II-J agar. Presence of juice favoured growth. CFU/mL were calculated from agar plugs recovered from CAMH-II agar and CAMH-II-J agar inoculated with CFT073.

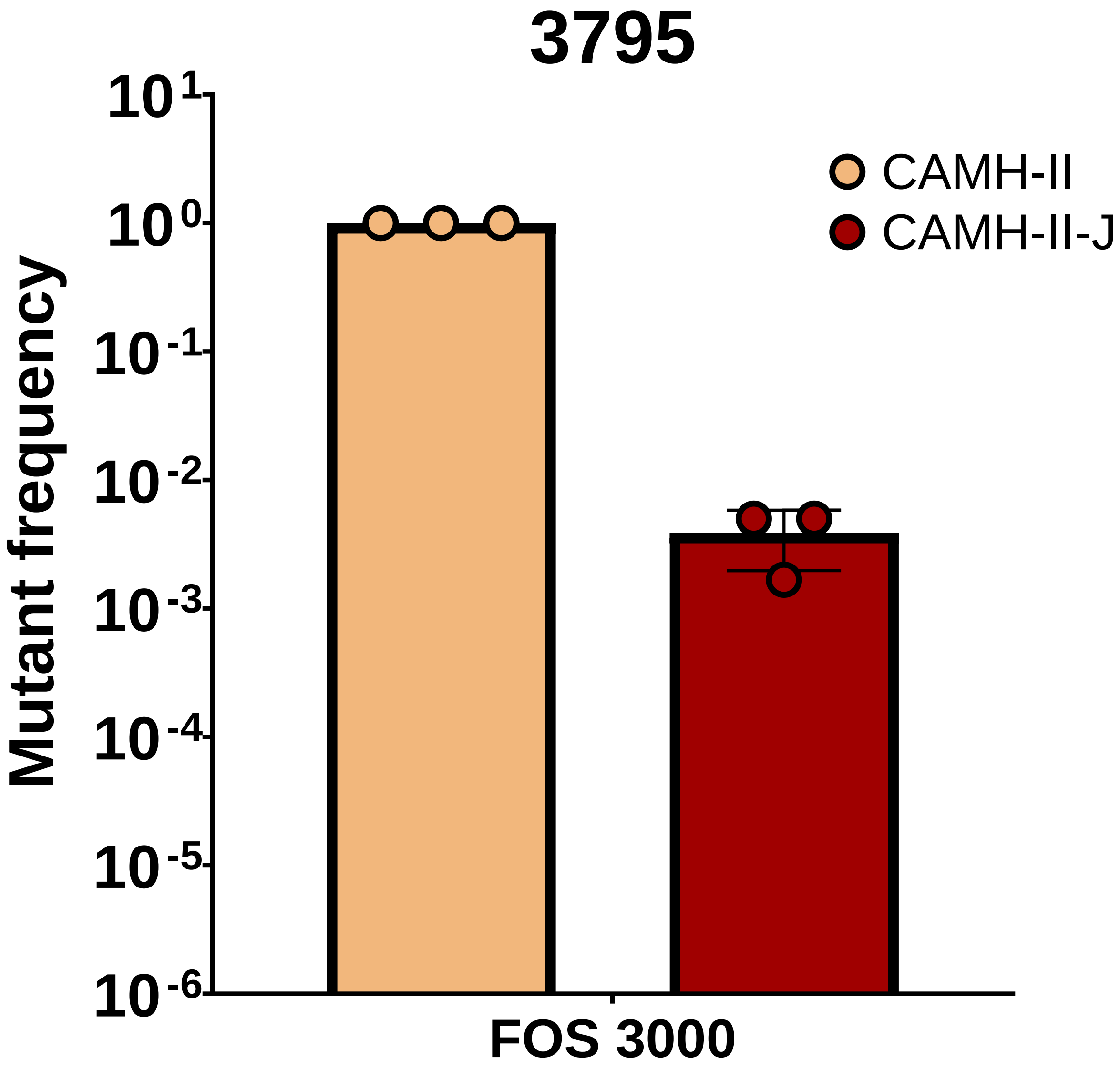

Figure S2: Mutant frequency of resistant strain 3795. Mutant frequency was calculated as the ratio between CFUs in presence of 3 mg/mL FOS and total CFUs in the absence of antibiotic. A ratio of 10^0 means CFU counts are equivalent between both conditions. Each dot represents an independent replicate. Experiment was performed at least twice.

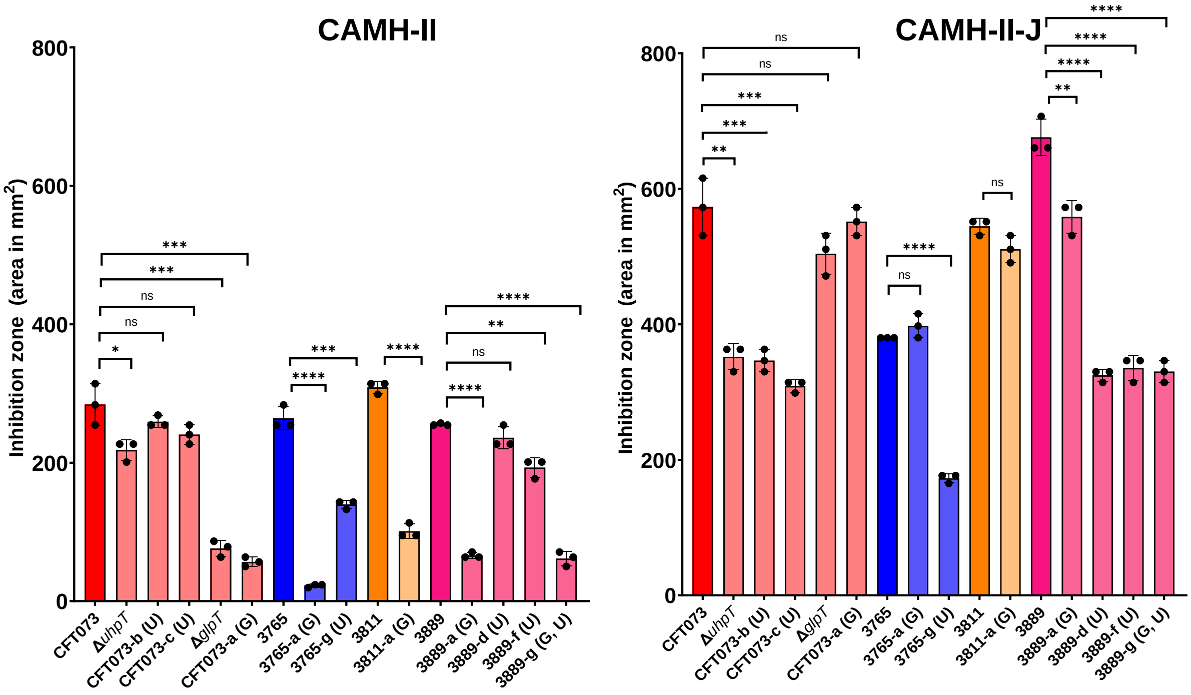

Figure S3: Comparison between inhibition zone areas on CAMH-II compared to CAMH-II-J. Inhibition zone areas were obtained in disk diffusion assays with 200 µg FOS on CAMH-II or CAMH-II-J agar after 24h incubation at 37°C for four selected UPEC *E. coli* strains and isolated FOS^R clones. (G) refers to isolates with impaired GlpT system and (U) refers to isolates with impaired UhpT system. This experiment was performed at least twice with similar results. Each dot represents a replicate. Errors bars represent standard variations obtained from three replicates. **** p<0.00005, *** P<0.0005, ** P<0.005, * P<0.05, ns: non-significant.

References

1. Guyomard-Rabenirina S, Malespine J, Ducat C, Sadikalay S, Falord M, Harrois D, Richard V, Dozois C, Laboratory working g, Breurec S, Talarmin A. 2016. Temporal trends and risks factors for antimicrobial resistant *Enterobacteriaceae* urinary isolates from outpatients in Guadeloupe. BMC Microbiol 16:121.
2. Mobley HL, Green DM, Trifillis AL, Johnson DE, Chippendale GR, Lockatell CV, Jones BD, Warren JW. 1990. Pyelonephritogenic *Escherichia coli* and killing of cultured human renal proximal tubular epithelial cells: role of hemolysin in some strains. Infect Immun 58:1281-9.
3. Welch RA, Burland V, Plunkett G, 3rd, Redford P, Roesch P, Rasko D, Buckles EL, Liou SR, Boutin A, Hackett J, Stroud D, Mayhew GF, Rose DJ, Zhou S, Schwartz DC, Perna NT, Mobley HL, Donnenberg MS, Blattner FR. 2002. Extensive mosaic structure revealed by the complete genome sequence of uropathogenic *Escherichia coli*. Proc Natl Acad Sci U S A 99:17020-4.
